# “I felt angry, but I couldn’t do anything about it”: a qualitative study of cyberbullying among Taiwanese high school students

**DOI:** 10.1186/s12889-019-7005-9

**Published:** 2019-05-28

**Authors:** Chia-Wen Wang, Patou Masika Musumari, Teeranee Techasrivichien, S. Pilar Suguimoto, Chang-Chuan Chan, Masako Ono-Kihara, Masahiro Kihara, Takeo Nakayama

**Affiliations:** 1 Department of Health Informatics, Kyoto University School of Public Health, Yoshida Konoe-Cho, Sakyo-Ku, Kyoto, 〒606-8501 Japan; 2 Interdisciplinary Unit for Global Health, Center for the Promotion of Interdisciplinary Education and Research, Kyoto University, Yoshida-honmachi, Sakyo-Ku, Kyoto, 〒606-8317 Japan; 3 Medical Education Center, Kyoto University Graduate School of Medicine, Yoshida Konoe-Cho, Sakyo-Ku, Kyoto, 〒606-8501 Japan; 4 Institute of Occupational Medicine and Industrial Hygiene, College of Public Health, National Taiwan University, No.17, Xu-Zhou Rd, Taipei, 10055 Taiwan

**Keywords:** Cyberbullying, Asian context, Social networking services, Qualitative research

## Abstract

**Background:**

Cyberbullying is a growing public health concern threatening the well-being of adolescents in both developed and developing countries. In Taiwan, qualitative research exploring the experiences and perceptions of cyberbullying among Taiwanese young people is lacking.

**Methods:**

We conducted in-depth interviews with a convenience sample of high school students (aged 16 to 18) from five schools in Taipei, Taiwan, without prior knowledge of their cyberbullying experiences. In total, 48 participants were interviewed.

**Results:**

We found that the experience of cyberbullying is common, frequently occurs anonymously and publicly on unofficial school Facebook pages created by students themselves, and manifests in multiple ways, such as name-calling, uploading photos, and/or excluding victims from online groups of friends. Exclusion, which may be a type of cyberbullying unique to the Asian context, causes a sense of isolation, helplessness, or hopelessness, even producing mental health effects in the victims because people place the utmost importance on interpersonal harmony due to the Confucian values in collectivistic Asian societies. In addition, our study revealed reasons for cyberbullying that also potentially reflect the collectivistic values of Asian societies. These reasons included fun, discrimination, jealousy, revenge, and punishment of peers who broke school or social rules/norms, for example, by cheating others or being promiscuous.

**Conclusions:**

Our findings reveal the pressing need for the Taiwanese school system to develop cyberbullying prevention programmes considering the nature and sociocultural characteristics of cyberbullying.

## Background

In recent years, with the rapid growth of information and communication technologies (ICTs), including the internet, social networking services (SNSs), and smartphones, a particular form of bullying referred to as *cyberbullying* has emerged. Past studies have documented the adverse health effects of traditional bullying on victims, including but not limited to psychosomatic problems [1], anxiety and depression [2], and suicidal ideation and suicidal behaviours [3]. Cyberbullying is often characterized by anonymity and publicity [4–6] and may result in significantly more negative consequences than traditional bullying. Past studies have suggested that victims of cyberbullying experienced more distress and had a higher risk of suicide ideation and attempts than victims of traditional bullying at school [7–9].

Asia, with approximately 4.2 billion people, has the largest population in the world and has been experiencing exponential growth of ICT usage during the last few decades. One statistical report documented that internet usage in Asia has increased 1670% since 2000 [10]. In particular, the overall penetration of internet usage has exceeded 80% of the population in certain countries, such as Hong Kong (87.0%), Japan (93.3%), South Korea (92.6%), and Taiwan (87.9%) [11]. In this context, the pervasiveness of ICT usage is alarming considering the urgent and critical issue of cyberbullying in Asian countries [12]. Although this issue has received little attention, the phenomenon has been found to be pervasive among adolescents in Asia. Studies from Taiwan, China, South Korea, and Japan have shown prevalence rates ranging from 6.3 to 34.8% for cyberbullying perpetration and from 14.6 to 56.9% for cyberbullying victimization [13–16]. These studies suggest that factors such as gender [13–15], electronic media (instant messaging, chat rooms, websites and bulletin board systems, e-mail, cell phones, SNSs, etc.) [13, 14], academic achievement [14], internet usage time [14, 15], and prior traditional bullying experiences [14, 15] are associated with cyberbullying.

Many studies on cyberbullying have been conducted in Western countries [5, 7, 17–21] using both qualitative and quantitative approaches, whereas research on cyberbullying in Asian regions [13–15, 22], whether qualitative or quantitative, remains scarce. Furthermore, past studies on cyberbullying in Asia have predominately been conducted using a quantitative approach to analyse the prevalence and related factors regarding cyberbullying, yet adolescents’ experiences and perceptions in the Asian context have not received much attention.

Cyberbullying is context-dependent, namely, influenced by the sociocultural environment [13]. Some studies have suggested that sociocultural factors should be considered to understand differences in the cyberbullying phenomenon between Asian and Western countries. For example, Shapka and Law (2013) found that ethnic differences between Canadian adolescents of East Asian and European descent were related to cyberbullying engagement [23]. Li (2008) found different patterns regarding cyberbullying experiences between Canadian and Chinese students, also suggesting that access to various ICTs may increase the risk of being involved in cyberbullying [24]. Furthermore, a short-term longitudinal study indicated cultural differences in cyberbullying between U.S. students and Japanese students [25].

A qualitative approach offers a useful means to explore the cyberbullying experiences of adolescents in the Asian social context in depth. This study employed a qualitative approach to explore the experiences and perceptions of cyberbullying among high school students in Taiwan.

## Methods

### Study design, participants, and setting

This is a qualitative study conducted between June and November 2016 using convenience sampling of high school students aged 16–18 from five high schools in Taipei, Taiwan. Participants in this study were recruited without prior knowledge of their cyberbullying experiences either as victims or perpetrators owing to the difficulties of identifying the victims and perpetrators of cyberbullying as indicated in previous studies [5, 21]. Teachers announced the interview opportunity in class to help recruit student volunteers. Given the sensitive nature of the topic of cyberbullying, the teachers did not mention the word “bullying” in the announcement. They mentioned only that the researchers wanted to interview students about their internet usage experiences. Subsequently, potential student volunteers contacted the teachers privately to obtain more details about the interview (namely, that the interview would address their opinions, perceptions and experiences regarding cyberbullying) to decide whether to participate. If the students and their legal guardians both agreed, then the researchers arranged an interview time. This study relied on voluntary participation. All participants and their guardians received information about the study’s purpose, its strict confidentiality, the voluntary nature of their participation, and their right to withdraw from the interview at any time. The participants and their guardians provided written informed consent prior to the interviews. Psychotherapy or mental health counselling was provided by the researcher during the study when requested by a participant. In addition, participants were referred to a hospital psychiatrist or clinical psychologist if they were found to be experiencing psychological distress or were identified as having severe suicidal ideation. We provided stationery and snacks to the students as tokens of appreciation for their time.

### Data collection and analysis

Data were collected through in-depth interviews guided by a semi-structured questionnaire. All interviews were audio-recorded and conducted in Mandarin by the same researcher (first author), and each interview lasted 30 to 100 min. The interviews were conducted in a designated room at each school that was occupied only by the researcher and participant to ensure the participants’ privacy and confidentiality. Prior to the interviews, the participants answered a short questionnaire including questions regarding sociodemographic characteristics (age, gender, etc.) and internet and ICT-related factors (internet usage time, tools to access the internet, etc.). The interviews explored the students’ experiences and perceptions of cyberbullying. Table 1 displays the topics and items included in the in-depth interviews.

**Table 1:** Interview items to explore the experiences and perceptions of cyberbullying

Topic	Contents of questions
Notion of cyberbullying	• Have you ever heard about cyberbullying? • What is cyberbullying?
Cyberbullying experiences as a bystander	• Have you ever witnessed cyberbullying during your high school life? • Who cyberbullied who? Who was the victim? • When and where did cyberbullying happen? How? • What were the reasons for cyberbullying? • What behaviours do you consider cyberbullying? Why?
Cyberbullying experiences as a victim	• Have you ever been cyberbullied? • Who cyberbullied you? • When and where did cyberbullying happen to you? How? • What do think were the reasons for being cyberbullied? • How did you react to the cyberbullying?
Cyberbullying experiences as a perpetrator	• Have you ever cyberbullied someone? • When and where did cyberbullying happen? How? • What do you think were the reasons for cyberbullying?

The interviews were transcribed verbatim and imported into QSR International’s Nvivo10 software. To perform the analyses, we used investigator triangulation and thematic analysis, an approach that involves familiarization with the data through an iterative process of reading the transcripts, generating codes, and arranging them into larger categorical groups (subcategories, categories, and themes) until a saturated thematic map of the data is obtained [26]. We revised and refined the themes until we achieved a consensus.

## Results

In total, 48 participants were interviewed [26 male students (54.2%) and 22 female students (45.8%)]. Most of the participants (77.1%) lived with both their parents, used a smartphone as a tool to access the internet (75.0%), and used the internet for at least 2 hours per day (66.7%) (Table 2).

**Table 2:** Demographic characteristics of the participants

	Male (*N* = 26)	%	Female (*N* = 22)	%	Total (*N* = 48)	%
Age						
16	5	19.2	6	27.3	11	22.9
17	13	50.0	11	50.0	24	50.0
18	8	30.8	5	22.7	13	27.1
Family situation						
Living with both parents	21	80.8	16	72.7	37	77.1
Living with a single parent	4	15.4	5	22.7	9	18.8
Living with others	1	3.8	1	4.5	2	4.2
Device most frequently used to access the Internet						
Desktop computer	7	26.9	1	4.5	8	16.7
Laptop	2	7.7	2	9.1	4	8.3
Smartphone	17	65.4	19	86.4	36	75.0
Internet usage time						
*School days (hours/per day)*						
less than 0.5	2	7.7	1	4.5	3	6.3
0.5 to < 1	2	7.7	1	4.5	3	6.3
1 to < 2	6	23.1	4	18.2	10	20.8
2 to < 3	7	26.9	7	31.8	14	29.2
3 to < 4	5	19.2	4	18.2	9	18.8
4 to < 5	2	7.7	3	13.6	5	10.4
5 or more	2	7.7	2	9.1	4	8.3
*Holidays (hours/per day)*						
less than 0.5	2	7.7	0	0.0	2	4.2
0.5 to < 1	0	0.0	0	0.0	0	0.0
1 to < 2	5	19.2	1	4.5	6	12.5
2 to < 3	4	15.4	7	31.8	11	22.9
3 to < 4	7	26.9	4	18.2	11	22.9
4 to < 5	4	15.4	4	18.2	8	16.7
5 or more	4	15.4	6	27.3	10	20.8

Total percentages may differ from 100 due to rounding

Of the 48 participants, 12 students (25.0%) reported a personal history of being a victim of cyberbullying, and the majority of the victims [10 of 12 (83.3%)] also reported being witnesses. The remainder of the students (75.0%) reported witnessing cyberbullying by friends, classmates, or schoolmates; however, none of them reported ever being a perpetrator. We identified six main themes, which are presented below along with supporting quotes. In some instances, the quotes were slightly edited for fluency.

### Theme 1: the sites of cyberbullying

Most participants [38 of 48 (79.2%)] reported that SNSs were the venues in which they were most likely to experience or witness cyberbullying, including unofficial school Facebook pages, personal Facebook pages, Instagram and Meteor (an SNS that is popular among Taiwanese high school students). In particular, they explained that cyberbullying often emerged on unofficial school Facebook pages. These pages are unrestricted and are created by students themselves to anonymously express their feelings or complaints concerning someone or something related to their school. One of the victims stated:

*“I saw that they verbally abused me on our unofficial school Facebook page, and many idiots (schoolmates) didn’t know the truth, and then, they clicked the ‘Like’ button on that post. I felt angry that they agreed with the perpetrators. I couldn’t do anything about it [angry face].” [16, M]*

Some participants [10 of 48 (20.8%)] also reported instances of cyberbullying such as uploading photos without approval through instant messaging applications such as LINE (a popular app in Taiwan for instant communication). One participant said:

*“She felt angry that her classmates downloaded her Facebook photos without permission and re-uploaded the photos without her approval to the LINE class group.” [17, F]*

A few of the participants [4 of 48 (8.3%)], particularly boys, indicated that online gaming, specifically multi-player or violent games, was another online context where they had witnessed or experienced cyberbullying. One victim said:

*“They [the online game players] verbally abused me because my performance was poor. Then, they would command you to change the online game character. If you did not follow their requests, they would attack you repeatedly. I felt very uncomfortable when I played the game.” [17, M]*

### Theme 2: the features of cyberbullying

In the interviews, the participants reported some features of cyberbullying, including anonymity, publicity, and permanency, which result in negative feelings such as anger or sadness.

### Anonymity

The majority of participants [32 of 48 (66.7%)] stated that cyberbullying was characterized by anonymity, indicating that perpetrators could attack victims but remain anonymous. According to the victims, nearly half of the victims [5 of 12 (41.7%)] stated that in their experience, they were cyberbullied anonymously. They mentioned that they felt powerless when being bullied online. This feeling was mostly related to the fact that the perpetrators were anonymous, precluding the victims from taking action to resolve the issue (for example, by removing inappropriate content from SNSs), as expressed in the following statements:

*“Someone attacked and verbally abused me online, and what he/she said was not the truth. It’s been hurtful to me. Things got worse, and some people believed what that person posted about me. I felt like I couldn’t defend myself, and whatever I said, people didn’t believe me.” [16, F]*

*“If the perpetrator is anonymous, you don’t know who he/she is, and you cannot ask him/her to delete the content [degrading photos or embarrassing videos].” [17, F]*

In addition, some of the participants [11 of 48 (22.9%)] mentioned how the perpetrators remained anonymous on social media sites. For example, Crush Ninja was popular among students for managing their own anonymous pages as well as public unofficial school Facebook pages to maintain anonymity or hide their IP addresses. One participant said:

*“They [the perpetrators] verbally abused someone on our unofficial school Facebook page. However, their names were not shown on that page. They submitted their posts to the third-party platform (CrushNinja), and then the posts were submitted by the third-party platform without revealing their identities.” [18, M]*

This study found that an anonymous social media site called Meteor is highly popular among Taiwanese high school students. On this site, perpetrators can attack victims without revealing their identities. One victim stated:

*“Someone verbally abused me and my friend on Meteor. I felt very hurt. The post was anonymous and did not show who posted the message. I didn’t know who attacked us.” [16, F]*

### Publicity

In addition, half of the participants [25 of 48 (52.1%)] frequently mentioned the public nature of cyberbullying, resulting in public exposure of the victims and easy engagement of other cyber bystanders as one of the participants described:

*“Sometimes, they [the perpetrators] directly write your student number, and your classmates will recognize you through your student number and tag you [on Facebook]. Then, they would verbally abuse you jointly.” [16, F]*

### Permanency

Some participants [12 of 48 (25.0%)] mentioned that they felt awful or hurt due to the permanency of cyberbullying on SNSs. From the victims’ perspective, some victims [4 of 12 (33.3%)] felt angry that they could not remove demeaning or embarrassing content themselves. Additionally, a few participants [5 of 48 (10.4%)] felt terrified that once posted online, the content would remain there forever. The participants stated:

*“I think our unofficial school Facebook page should be removed. Someone called me names on it. I felt very uncomfortable [angry face].” [18, F]*

*“The posts on our unofficial school Facebook page would remain online forever. Even if you later felt sorry about attacking the victims, you couldn’t withdraw what you posted.” [16, F]*

*“One of my classmates wanted to remove what she had posted on our unofficial school Facebook page. Although she contacted the manager of our unofficial school Facebook page, the manager did not remove the post.” [16, F]*

### Theme 3: the types of cyberbullying

The participants reported that the most common type of cyberbullying was name-calling (gossiping) [38 of 48 (79.2%)], followed by posting photos [12 of 48 (25.0%)] and exclusion (isolation) [4 of 48 (8.3%)], as shown in the following statements:

### Name-calling (gossiping)

*“They [the perpetrators] created two accounts on Instagram. One was open to the public, and the other one was privately shared between a few good friends. They used the private account to gossip and call other classmates or schoolmates names.*” *[17, F]*

*“She gossiped about me on her private Instagram account, and one of my classmates who followed her account took a screenshot of the malicious gossip and forwarded it to me.” [16, F]*

### Posting photos

*“I once witnessed someone intentionally posting a girl’s photo using an anonymous account on our unofficial school Facebook page. He [or she] took the photo of the girl, uploaded it, and verbally abused her. I felt like s/he [the perpetrator] intentionally did it to hurt the girl.” [17, F]*

### Exclusion (isolation)

The participants reported that to isolate them, perpetrators would exclude victims by creating a group on LINE that included all their classmates except for the victims. The participants stated:

*“He is very bai-mu [a slang term in the local Taiwanese language used to describe an individual who does not understand a situation and then engages in inappropriate behaviour to annoy other people], so classmates dislike him, and he is not in our LINE class group; none of our classmates have included him in the group, although sometimes important class announcements are posted on the group [without informing him].” [17, F]*

*“Well, a girl was rude, so our classmates disliked her. They created a group (on LINE) to speak ill of her. All our classmates were included in that group except for her. I was also included in that group, although I didn’t want to be. However, if I quit the group, it would be like I was on her side. So, I didn’t know what to do.” [17, F]*

### The overlap with traditional bullying

Although we did not explicitly ask about traditional bullying, we found an overlap between cyberbullying and traditional bullying. Some of the victims [4 of 12 (33.3%)] of cyberbullying also reported having experienced traditional bullying at school. They reported that they felt sad for being bullied not only at school but also on the internet. One of the victims stated:

*“When I was walking over, they [the classmates] called me bitch, and they often gossiped about me. I couldn’t do anything because no one stood by my side [sad face]. If I fought back, they would attack me even more aggressively…Someone [publicly] insulted me [on Meteor, a highly popular SNS among Taiwanese high school students] and gossiped that I had sex with someone and called me a bitch.” [16, F]*

### Theme 4: motivation for cyberbullying

The participants mentioned several reasons for cyberbullying, including fun, punishment, discrimination, jealousy, and revenge.

### For fun

Nearly half of the participants [23 of 48 (47.9%)] reported that the most common reason for cyberbullying was “*for entertainment or for fun.*” One participant stated:

*“They felt that it was fun to post his [a classmate with emotional disorders] videos on the Facebook page.” [18, M]*

### For punishment

Some participants [15 of 48 (31.3%)] reported that other schoolmates (or classmates) were annoyed because the victims did something wrong at school, such as cheating or being sexually promiscuous, or the victims were rude or *bai-mu*, which is why the victims were then bullied. The participants stated:

*“A girl in our class was verbally abused on our unofficial school Facebook page because she cheated on an exam. She was depressed for a long time.” [17, F]*

*“A girl was repeatedly attacked on our unofficial school Facebook page because she was hooking up with many guys at our school, and her real name was posted openly.” [18, M]*

*“I saw that a schoolmate’s name was posted and that he was verbally abused on our unofficial school Facebook page. I knew him because we were classmates in 10* ^*th*^ *grade. He is bai-mu and obnoxious. Many people hate him, including me.” [18, M]*

### For revenge

Revenge as a reason for cyberbullying was mentioned by a few participants [5 of 48 (10.4%)]. For example, one of the participants described an incident of cyberbullying that occurred in her class. A victim of traditional bullying could not tolerate his perpetrator’s constant teasing of him in class, and the victim therefore took revenge on the perpetrator online. The participant stated:

*“The boy thought that it was very funny to tease him [the victim]. In the beginning, I thought that it was funny, too. However, he made fun of him almost every class. It turned out that XXX [the victim’s name] anonymously verbally abused the boy who always made fun of him on our unofficial school Facebook page.” [16, F]*

### For discrimination

In a few instances [3 of 48 (6.3%)], minorities (sexual minorities and disabled students) at school were the targets of cyberbullying. Participants reported the following:

*“I have been insulted [on Facebook Messenger] by my schoolmates because I’m homosexual. They called me the lady boy and told me that I’m disgusting.” [17, F]*

*“We created a specific page for him [a student with emotional disorders] on Facebook to post his behaviours. [He (the victim)] cannot control his emotions... sometimes a video in which he was shouting was posted....” [18, M]*

### From jealousy

A few participants [2 of 48 (4.2%)] mentioned that some of the perpetrators were jealous of the victims’ success in sports or academics as one of the participants described:

“*Not only was he an athlete on the national team but his academic performance was also excellent. Some schoolmates felt that he was up on a high horse. So, they attacked him on our unofficial school Facebook page.*” *[17, F]*

### Theme 5: ambiguity and context dependency

The notion of cyberbullying was not clear to many of the participants, which caused confusion regarding whether certain behaviours would be considered cyberbullying. Many participants [26 of 48 (54.2%)] found distinguishing between cyberbullying and “*just having fun*” on LINE or other SNSs difficult. This difficulty is illustrated in the following quotes:

*“They posted my photo as the cover photo of our LINE class group, but I did not care because I thought they were just kidding.” [17, M]*

*“He [an unfamiliar classmate] uploaded my photo, and I didn’t like it. I’m not sure whether this behaviour could be called cyberbullying.” [18, M]*

In addition, the participants mentioned that whether a particular behaviour would be considered cyberbullying was based on the nature of the relationship of the involved students. They argued that between good friends, actions are interpreted as jokes, but these actions would be perceived as cyberbullying attacks if they came from unfamiliar people. For example, the participants explained:

*“My sleeping photos have often been posted as the cover photos of our LINE class group since the 10* ^*th*^ *grade. However, I do not care. I know that they are kidding rather than trying to hurt me. Additionally, the classmates who always post my photos have a good relationship with me, so I feel that it’s OK. If unfamiliar people [classmates or schoolmates] post my photos, I will demand that they remove the photos. It depends on the relationship with that person [to differentiate between jokes and cyberbullying].” [18, F]*

*“They uploaded my photos on the LINE group. We were good friends, so I felt very amused. I thought they were just kidding.” [16, M]*

### Theme 6: coping strategies of victims

Coping with cyberbullying seemed difficult; half of the victims [6 of 12 (50.0%)] reported that they ignored the bullying. However, some of the victims reported coping strategies, including talking with friends, expecting teachers to intervene, confrontation, and leaving the group.

### Ignoring cyberbullying/taking no action

Half of the victims [6 of 12 (50.0%)] reported that they ignored cyberbullying or took no action when they experienced cyberbullying.

*“They verbally abused me on our unofficial school Facebook page. I thought that they had nothing better to do and I just ignored it [cyberbullying].” [18, F]*

*“I felt angry, but I couldn’t do anything about it [cyberbullying] since he/she remained anonymous. I could not figure out who attacked me.” [17, F]*

### Talking with friends

Three of the 12 victims (25.0%) talked with friends to express their feelings. One victim said:

*“I felt very angry, but I couldn’t do anything about it. The one thing that I could do was talk to my friends. My friends comforted me and told me not to take it so seriously.” [18, F]*

### Expecting teachers to intervene

In a few instances [2 of 12 (16.7%)], the victims explained that responding to cyberbullying was difficult due to the anonymity of the perpetrators and expressed the hope that teachers could identify the perpetrators. However, they felt that teachers could not address cyberbullying since the perpetrators remained anonymous. One participant described the following:

*“I think that the teachers should deal with cyberbullying. However, the teachers may not be able to find out who the perpetrator is due to anonymity.” [18, F]*

### Confrontation

In a few cases [2 of 12 (16.7%)] where the victim knew the identity of the perpetrator, some victims felt angry or hurt and confronted the perpetrator(s) to demand the removal of demeaning content from SNSs. A victim stated:

“*He [the classmate] uploaded my photo as his Facebook profile picture, but I demanded that he remove my photo.” [18, M]*

### Leaving the group

Only one of the 12 victims (8.3%) mentioned she left a chat group in response to cyberbullying. She said:

“*They [the schoolmates] were gossiping about me on the chat group on Facebook Messenger, but I didn’t reply to the message and quit the chat group.” [17, F]*

Table 3 displays the percentage representations of the six themes.

**Table 3:** Percentage of victims or participants indicating specific themes related to cyberbullying

Themes	N of participants or victims	% of participants or victims
The sites of cyberbullying		
SNSs	38	79.2
Instant messaging applications	10	20.8
Multiplayer online games	4	8.3
The features of cyberbullying		
Anonymity	32	66.7
Publicity	25	52.1
Permanency	12	25.0
The types of cyberbullying		
Name-calling (gossiping)	38	79.2
Posting photos	12	25.0
Exclusion (isolation)	4	8.3
Overlap with traditional bullying	4 (victims)	33.3^a^
Motivation for cyberbullying		
For fun	23	47.9
For punishment	15	31.3
For revenge	5	10.4
For discrimination	3	6.3
From jealousy	2	4.2
Ambiguity and context dependency		
Difficulties in distinguishing between cyberbullying and having fun	26	54.2
Coping strategies of victims		
Ignoring/no action	6 (victims)	50.0^a^
Talking with friends	3 (victims)	25.0^a^
Expecting teachers to intervene	2 (victims)	16.7^a^
Confrontation	2 (victims)	16.7^a^
Leaving the group	1 (victim)	8.3^a^

Some respondents (either victims or participants) responded more than once and therefore appear twice

^a^Percentage of the victims

## Discussion

To our knowledge, this is the first qualitative study to explore cyberbullying among Taiwanese high school students. Most previous studies have used a quantitative approach [13, 22, 27]. However, due to the complexity and sensitivity of cyberbullying, quantitative studies may not fully capture the breadth and depth of the problem.

From the results, we found some similarities and differences between Asian and Western contexts. Regarding the sites of cyberbullying, similar to Western societies [28, 29], cyberbullying predominantly occurs through SNSs. However, our study highlighted that students consistently mentioned cyberbullying experienced or witnessed on their unofficial school Facebook pages, which has rarely been reported in other studies. In Taiwan, many high school students have created unofficial school Facebook pages to express their feelings or complaints concerning someone or something at school. The anonymity and publicity [6, 30] of such sites were utilized to provide a cover for insults, humiliation, personal attacks, or assaults, allowing many cyber bystanders to attack victims jointly. The anonymity and publicity of cyberbullying, together with its permanency, create serious negative consequences that may cause long-term psychological effects for cyber victims.

With respect to the types of cyberbullying, name-calling (gossiping), posting photos, and an overlap with traditional bullying have also been reported in the Western context [18, 31–34]. In this study, we found that students used SNSs (Instagram) to gossip or call other people names, implying that they may learn about name-calling (gossiping) via Instagram as victims or bystanders. We recommend that future studies should address this issue to clarify whether students are actively participating in cyberbullying.

In addition, we found that group exclusion was very common, as reported in other Asian societies [14, 35, 36]. This study found that students used group exclusion to isolate a victim, for example, by creating a LINE group including everyone except for the victim(s). Previous studies from China and Hong Kong have documented group exclusion, including the use of online text to socially isolate victims [35] or kicking someone out of a chat room [14]. Such exclusion may cause feelings of isolation, helplessness, or hopelessness, producing mental health effects in victims of cyberbullying because people place the utmost importance on interpersonal harmony and a sense of belonging due to the Confucian values in collectivistic Asian societies [13, 37, 38].

Regarding the motivations for cyberbullying, fun [39], discrimination [40, 41], jealousy [42], and revenge [39, 41–43] were consistent with previous studies in Western societies. In addition, we found that punishment may be a significant motivation to cyberbully peers who break school rules, such as cheating, or social norms, such as traditional heterosexual roles [44] in Asian societies. In particular, group conformity is an important social rule in Asian society [38]; in this study, if students did something wrong or were different from others, as in the case of sexual minorities, they were easily targeted by other students.

In this study, we found that cyberbullying is ambiguous or highly context-dependent in Asian countries. Previous Western studies [20, 45] have mentioned “intention” as a critical criterion to distinguish cyberbullying from cyber jokes. However, our study showed that the distinction between cyberbullying and conventional jokes and pranks between friends was not clear to many students. Judgments regarding whether a particular act or behaviour could be considered cyberbullying were based on the closeness to or the nature of the relationship with the perpetrator. Therefore, most behaviours, however offensive, would be regarded as a joke or “*just for fun*” if they were performed by someone close because participants felt that such behaviours were not performed with the intent to hurt someone. This observation may explain why many high school students mentioned that cyberbullying was carried out for entertainment or fun. We suggest that in addition to the intention of the perpetrator, his or her relationship with peers can be used to define cyberbullying among adolescents in the Asian context. Additionally, power imbalance is an essential criterion for defining cyberbullying [45, 46]. Perpetrators may expose victims publicly, issuing psychological threats and causing the victims to feel powerless in the face of the potential cyber audience (based on the number of comments, likes, and shares) [47].

Regarding coping strategies, consistent with one study in China, most victims reported that they ignored the attacks [14]. This behaviour may indicate that passive coping strategies are predominantly adopted in Asian societies because these societies value interpersonal harmony and tolerance due to the social rules in relationships, again implying the core Confucian values in Asian contexts.

In contrast, active coping strategies, such as attempting to resolve problems or blocking a bully, have been commonly reported in Western countries [32, 48].

Although this study provided some insight into Taiwanese students’ experiences and perceptions of cyberbullying, we need to acknowledge some limitations. First, despite our efforts to ensure privacy during the interview, place participants at ease, and maintain strict confidentiality, students were reluctant to report being victims or perpetrators of cyberbullying (in the interviews, we found that a few participants initially spoke in the third person. However, they later spoke in the first person to disclose their stories). Due to the sensitive nature of the topic and the social desirability effect, we may have failed to capture some important aspects of cyberbullying in this study, especially the cyber perpetrators’ perspective. Second, voluntary participation may have introduced a self-selection bias.

## Conclusions

The experience of cyberbullying appears to be common among high school students and occurs in multiple forms (name-calling, posting photos, exclusion from online groups, etc.) and on multiple platforms (Facebook and instant messaging applications). Our findings underscore the pressing need for the Taiwanese school system to take action to prevent and stop cyberbullying, including developing students’ and teachers’ skills and appropriate response strategies, considering the nature of cyberbullying and sociocultural characteristics in Taiwan.

## Data Availability

This study is based on qualitative data, including observation field notes and interview transcripts. The participants did not consent to have their full transcripts shared publicly.

## Abbreviations

ICTs: Information and communication technologies
SNSs: Social networking services

## References

[1] Gini G, Pozzoli T (2009). Association between bullying and psychosomatic problems: a meta-analysis. Pediatrics.

[2] Kaltiala-Heino R, Rimpelä M, Rantanen P, Rimpelä A (2000). Bullying at school—an indicator of adolescents at risk for mental disorders. J Adolesc.

[3] Kim YS, Leventhal B (2008). Bullying and suicide. A review. Int J Adolesc Med Health.

[4] Patchin JW, Hinduja S (2006). Bullies move beyond the schoolyard a preliminary look at cyberbullying. Youth Violence Juvenile Justice.

[5] Slonje R, Smith PK (2008). Cyberbullying: another main type of bullying?. Scand J Psychol.

[6] Sticca F, Perren S (2013). Is cyberbullying worse than traditional bullying? Examining the differential roles of medium, publicity, and anonymity for the perceived severity of bullying. J Youth Adolesc.

[7] Schneider SK, O'donnell L, Stueve A, Coulter RW (2012). Cyberbullying, school bullying, and psychological distress: a regional census of high school students. Am J Public Health.

[8] Wang J, Nansel TR, Iannotti RJ (2011). Cyber and traditional bullying: differential association with depression. J Adolesc Health.

[9] Van Geel M, Vedder P, Tanilon J (2014). Relationship between peer victimization, cyberbullying, and suicide in children and adolescents: a meta-analysis. JAMA Pediatr.

[10] Internet World Stats (2018). World Internet Users and 2018 Population Stats.

[11] Internet World Stats. Asia internet use, population data and facebook statistics. 2018. https://www.internetworldstats.com/stats3.htm%23asia. Accessed 23Apr 2018.

[12] Bhat C, Ragan M (2013). Cyberbullying in Asia.

[13] Y-y H, Chou C (2010). An analysis of multiple factors of cyberbullying among junior high school students in Taiwan. Comput Human Behav..

[14] Zhou Z, Tang H, Tian Y, Wei H, Zhang F, Morrison CM (2013). Cyberbullying and its risk factors among Chinese high school students. Sch Psychol Int.

[15] Lee C, Shin N (2017). Prevalence of cyberbullying and predictors of cyberbullying perpetration among Korean adolescents. Comput Human Behav.

[16] Udris R (2014). Cyberbullying among high school students in Japan: development and validation of the online Disinhibition scale. Comput Human Behav..

[17] Hinduja S, Patchin JW (2010). Bullying, cyberbullying, and suicide. Arch Suicide Res.

[18] Smith PK, Mahdavi J, Carvalho M, Fisher S, Russell S, Tippett N (2008). Cyberbullying: its nature and impact in secondary school pupils. J Child Psychol Psychiatry.

[19] Elgar FJ, Napoletano A, Saul G, Dirks MA, Craig W, Poteat VP, Holt M, Koenig BW (2014). Cyberbullying victimization and mental health in adolescents and the moderating role of family dinners. JAMA Pediatr.

[20] Vandebosch H, Van Cleemput K (2008). Defining cyberbullying: a qualitative research into the perceptions of youngsters. CyberPsychol Behav.

[21] Mishna F, Saini M, Solomon S (2009). Ongoing and online: children and youth's perceptions of cyber bullying. Child Youth Serv Rev.

[22] Chang FC, Lee CM, Chiu CH, Hsi WY, Huang TF, Pan YC (2013). Relationships among cyberbullying, school bullying, and mental health in Taiwanese adolescents. J Sch Health.

[23] Shapka JD, Law DM (2013). Does one size fit all? Ethnic differences in parenting behaviors and motivations for adolescent engagement in cyberbullying. J Youth Adolesc..

[24] Li Q (2008). A cross-cultural comparison of adolescents' experience related to cyberbullying. Educ Res.

[25] Barlett CP, Gentile DA, Anderson CA, Suzuki K, Sakamoto A, Yamaoka A, Katsura R (2014). Cross-cultural differences in cyberbullying behavior: a short-term longitudinal study. J Cross-Cult Psychol.

[26] Braun V, Clarke V (2006). Using thematic analysis in psychology. Qual Res Psychol.

[27] Chang F-C, Chiu C-H, Miao N-F, Chen P-H, Lee C-M, Chiang J-T, Pan Y-C (2015). The relationship between parental mediation and internet addiction among adolescents, and the association with cyberbullying and depression. Compr Psychiatry.

[28] Dredge R, Gleeson J, de la Piedad Garcia X (2014). Cyberbullying in social networking sites: an adolescent victim’s perspective. Comput Human Behav.

[29] Sampasa-Kanyinga H, Hamilton HA (2015). Use of social networking sites and risk of cyberbullying victimization: a population-level study of adolescents. Cyberpsychol Behav Soc Netw.

[30] Dredge R, Gleeson JF, de la Piedad Garcia X (2014). Risk factors associated with impact severity of cyberbullying victimization: a qualitative study of adolescent online social networking. Cyberpsychol Behav Soc Netw.

[31] Sourander A, Klomek AB, Ikonen M (2010). Psychosocial risk factors associated with cyberbullying among adolescents: a population-based study. Arch Gen Psychiatry.

[32] Price M, Dalgleish J. Cyberbullying: Experiences, impacts and coping strategies as described by Australian young people [online]. Youth Stud Aust. 2010;29(2):51–9. Availability:<https://search.informit.com.au/documentSummary;dn=213627997089283;res=IELHSS>ISSN: 1038-2569. [cited 15 Jul 18].

[33] Juvonen J, Gross EF (2008). Extending the school grounds?—bullying experiences in cyberspace. J Sch Health..

[34] Kowalski RM, Morgan CA, Limber SP (2012). Traditional bullying as a potential warning sign of cyberbullying. Sch Psychol Int.

[35] Wong DS, Chan HCO, Cheng CH (2014). Cyberbullying perpetration and victimization among adolescents in Hong Kong. Child Youth Serv Rev.

[36] Rao Jiaming, Wang Haiqing, Pang Minhui, Yang Jianwei, Zhang Jiayi, Ye Yunfeng, Chen Xiongfei, Wang Shengyong, Dong Xiaomei (2017). Cyberbullying perpetration and victimisation among junior and senior high school students in Guangzhou, China. Injury Prevention.

[37] Triandis HC (2001). Individualism-collectivism and personality. J Pers.

[38] Kim H, Markus HR (1999). Deviance or uniqueness, harmony or conformity? A cultural analysis. J Pers Soc Psychol.

[39] Raskauskas J, Stoltz AD (2007). Involvement in traditional and electronic bullying among adolescents. Dev Psychol.

[40] Hoff DL, Mitchell SN (2009). Cyberbullying: causes, effects, and remedies. J Educ Adm.

[41] Varjas K, Meyers J, Kiperman S, Howard A (2013). Technology hurts? Lesbian, gay, and bisexual youth perspectives of technology and cyberbullying. J Sch Violence.

[42] Varjas K, Talley J, Meyers J, Parris L, Cutts H (2010). High school students’ perceptions of motivations for cyberbullying: an exploratory study. West J Emerg Med.

[43] König A, Gollwitzer M, Steffgen G (2010). Cyberbullying as an act of revenge?. J Psychol Couns Sch.

[44] Navarro R, Navarro R, Yubero S, Larrañaga E (2016). Gender issues and cyberbullying in children and adolescents: from gender differences to gender identity measures. Cyberbullying across the globe.

[45] Menesini E, Nocentini A, Palladino BE, Frisén A, Berne S, Ortega-Ruiz R, Calmaestra J, Scheithauer H, Schultze-Krumbholz A, Luik P (2012). Cyberbullying definition among adolescents: a comparison across six European countries. Cyberpsychol Behav Soc Netw.

[46] Langos C (2012). Cyberbullying: the challenge to define. Cyberpsychol Behav Soc Netw.

[47] Menesini E, Nocentini A, Palladino BE, Völlink T, Dehue F, Mc Guckin C (2016). Cyberbullying: conceptual, theoretical and methodological issues. Cyberbullying: from theory to intervention.

[48] Livingstone S, Haddon L, Görzig A, Ólafsson K (2011). Risks and safety on the internet: the perspective of European children: full findings and policy implications from the EU kids online survey of 9–16 year olds and their parents in 25 countries.

